# Case Report: Primary leiomyosarcoma of the canine gallbladder with intraoperative indocyanine green near-infrared fluorescence imaging

**DOI:** 10.3389/fvets.2025.1678285

**Published:** 2025-10-24

**Authors:** Jongchul Yun, Yujin Kim, Kyung-Mee Park, Sungin Lee

**Affiliations:** ^1^Department of Veterinary Surgery, College of Veterinary Medicine, Chungbuk National University, Cheongju, Republic of Korea; ^2^Laboratory of Veterinary Surgery & Ophthalmology, College of Veterinary Medicine, Chungbuk National University, Cheongju, Republic of Korea

**Keywords:** canine, gallbladder, leiomyosarcoma, indocyanine green, near-infrared fluorescence

## Abstract

A 13-year-old castrated male, Maltese was presented for abnormal findings of gallbladder on abdominal ultrasonography without clinical signs. Abdominal ultrasonography revealed a severely distended gallbladder and a heterogeneous echogenic mass in the gallbladder neck. No evidence of metastasis was observed. Cholecystectomy was performed with indocyanine green near-infrared fluorescence imaging for real-time visualization of the biliary tract that contributed to improve surgical outcomes. In the histopathological examination and immunohistochemical analysis using smooth muscle actin staining, the gallbladder mass was confirmed as a leiomyosarcoma. The patient has been followed up for 18 months without any signs of recurrence or metastasis. This is the first reported case of gallbladder leiomyosarcoma in dogs. Leiomyosarcoma should be considered a differential diagnosis for dogs with gallbladder mass. The histologic low grading, the absence of microscopic residual tumor and metastasis relate to good prognosis.

## 1 Introduction

Primary gallbladder sarcoma is a rare malignant tumor that arises from mesenchymal cells and represents 1.5% of human gallbladder malignancies. In a study of the reported cases of primary gallbladder sarcoma, leiomyosarcoma is a significant subtype accounting for 43% (28 of 65 cases) (1). Although radical cholecystectomy is considered a potentially effective treatment option, the prognosis of gallbladder leiomyosarcoma remains poor due to rapid progression (2). Complete surgical resection is significant prognostic factor, as the overall 5-year survival rate for gallbladder cancer in human patients with histologically confirmed complete surgical margins ranges from 21–69%, whereas patients with incomplete surgical margins have a reported survival rate of 0% (3).

Gallbladder tumors are rarely reported in veterinary medicine, of which neuroendocrine carcinomas are the most diagnosed (4). Other tumors reported include adenocarcinoma, adenoma, leiomyoma, lymphoma, and metastatic gastrointestinal stromal tumors (4). Early diagnosis of gallbladder tumors is challenging because patients rarely present with specific clinical signs unless biliary tract obstruction, rupture, hemorrhage, or localized invasion occurs (3).

Indocyanine green (ICG) is an amphiphilic tricarboncyanine iodide dye widely used for near-infrared fluorescence (NIRF) imaging to provide real-time visualization during surgical procedures. After intravenous injection, ICG binds to plasma proteins and is distributed intravascularly until selective uptake by hepatocytes and excretion into the bile without metabolic transformation (5). Based on this pharmacokinetics, ICG has been used for hepatic function tests and measurement of hepatic blood flow in humans (6). Recently, clinical applications have expanded to evaluation of liver perfusion (7), segmental mapping (8), intraoperative cholangiography (9), and sentinel lymph node detection (10). In veterinary medicine, pilot study has demonstrated the feasibility of ICG NIRF imaging for intraoperative cholangiography (11) and related applications (12, 13). However, its clinical use remains limited.

This report describes the first documented case of primary gallbladder leiomyosarcoma in a dog. The aim of the report is to discuss the clinical presentation, diagnostic process, management, prognostic implications of this rare condition, and to demonstrate the feasibility of ICG NIRF in cholecystectomy.

## 2 Case description

A 13-year-old, castrated male, 4.77 kg, Maltese was referred for the finding of internal septation in gallbladder on abdominal ultrasonography. The patient had no clinical signs related to gallbladder disease.

**Figure 1:**
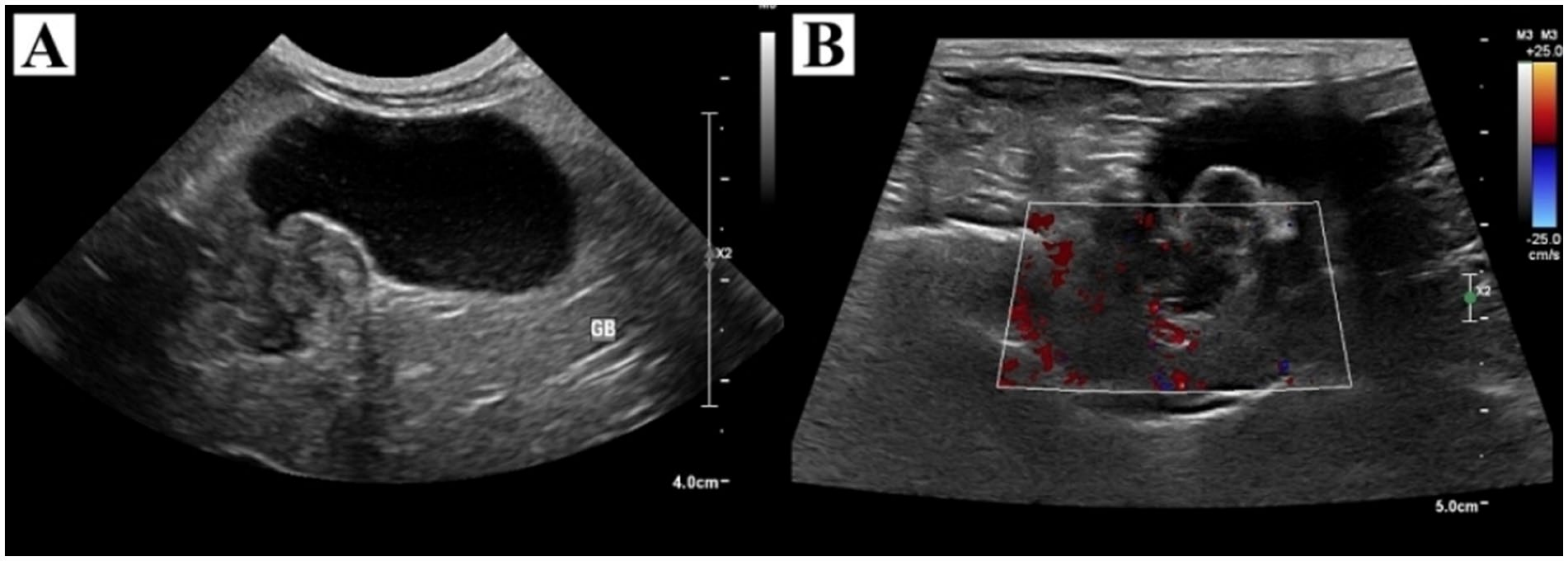
Ultrasonographic findings of the gallbladder and tumor. **(A)** The gallbladder was severely distended with heterogeneous echogenic material in the neck and had no concurrent gallbladder wall thickening. **(B)** Color Doppler imaging revealed no distinct vascularity within the mass.

Physical examination revealed no abnormalities. Complete blood cell count, blood gas analysis, and coagulation tests showed no clinically significant abnormalities. Serum biochemistry revealed elevations in alkaline phosphatase (573 IU/L, reference range, 29–97 IU/L), aspartate transaminase (101 IU/L, reference range, 23–66 IU/L), gamma-glutamyl transferase (12 IU/L, reference range, 1–10 IU/L), and lactate (3.51 mmol/L, reference range, 0.5–2.5 mmol/L). Thoracic and abdominal radiographs revealed no abnormalities. On abdominal ultrasonography, the gallbladder was severely distended. The neck of the gallbladder appeared 1.7 × 1.5 cm, sessile, tortuous, and a heterogeneous echogenic mass was identified (Figure 1). Color Doppler imaging revealed no distinct vascularity within the mass. The common bile duct was mildly dilated. There was no evidence of metastasis on abdominal ultrasound. Preoperative CT was not performed due to the owner’s financial constraint.

Based on imaging findings, gallbladder tumors such as neuroendocrine carcinoma, adenocarcinoma, leiomyoma, adenoma, and lymphoma were primarily considered. However, due to overlapping ultrasonographic imaging features, partial extrahepatic biliary obstructions caused by bile duct plugs or non-gravity-dependent sludge were also considered.

**Figure 2:**
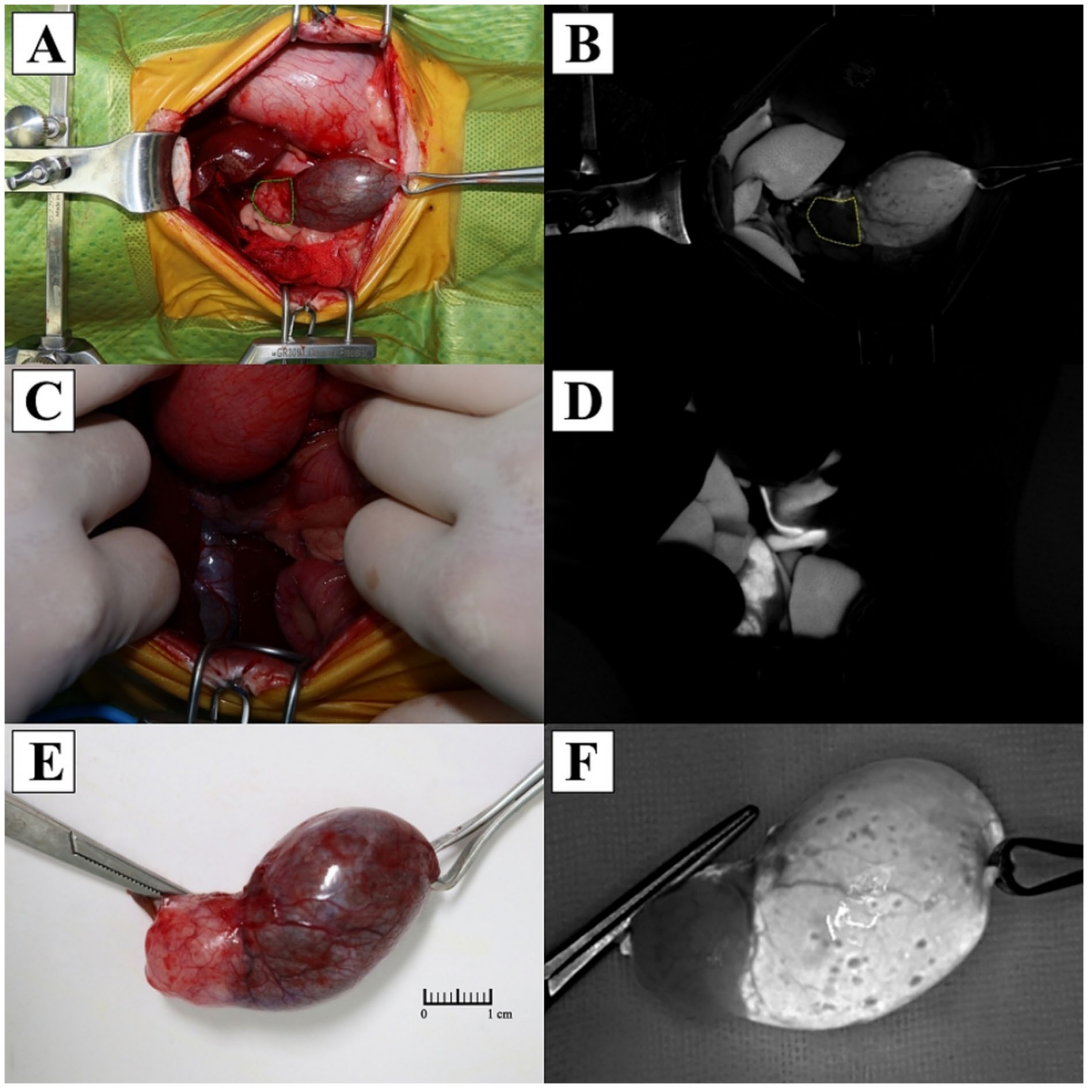
Comparison of standard views and indocyanine green near-infrared fluorescence imaging during cholecystectomy. **(A)** Intraoperative standard view of the gallbladder and tumor (dotted green line) following dissection. **(B)** Indocyanine green fluorescence of the filling defect at the tumor site (dotted yellow line). **(C)** Intraoperative standard view of the biliary tract. **(D)** Indocyanine green fluorescence in the biliary tract. **(E)** Gross specimen of the resected gallbladder. **(F)** Near-infrared fluorescence imaging of the resected gallbladder using indocyanine green.

Cholecystectomy using ICG NIRF imaging was performed along with liver biopsy to evaluate for other hepatic diseases (Figure 2). The patient was premedicated with cefazolin (22 mg/kg, IV; Cephazolin sodium, Chong Kun Dang Pharmaceutical Corporation., Seoul, Korea), famotidine (1 mg/kg, IV; Gaster inj., Dong-A Pharm, Seoul, Korea), and midazolam (0.2 mg/kg IV; Bukwang midazolam inj., Bukwang Pharm, Seoul, Korea). ICG (0.05 mg/kg, IV; Cellbiongreen inj., Cellbion, Seoul, Korea) was injected 45 min before surgery to visualize the biliary tract. No adverse reaction associated with ICG administration was observed. Anesthesia was induced with propofol (8 mg/kg, IV; Freepol-MCT Inj., Daewon Pharm, Seoul, Korea). General anesthesia was maintained with isoflurane in 100% oxygen. Perioperative analgesia was provided with a continuous rate infusion of fentanyl (2–9 mcg/kg/h; Fentanyl Citrate Inj., Hana Pharm, Seoul, Korea).

The patient was positioned in dorsal recumbency, and a ventral midline incision was made from the xiphoid process extending caudally to the pubis. The distended gallbladder and dilated common bile duct were identified. Intraoperative cholangiography was performed using ICG NIRF imaging system (ZNI; Metaple Bio Co., Ltd., Seoul, Korea) which consists of a control unit and an NIR light source emitting at 802.5 nm. The working distance between the light source and the surgical site was maintained at approximately 40 cm, and the operating room lights were turned off to optimized fluorescence visualization. ICG fluorescence revealed partial filling defects in the gallbladder at the site of the tumor, but fluorescence was clearly visualized in the common bile duct indicating patency. After confirming patency, the gallbladder was dissected from the hepatic fossa using blunt dissection with sterilized cotton swabs and electrocautery. After ligation of the cystic duct and artery with two non-absorbable, polymer locking clips, and 3–0 absorbable suture, the gallbladder was completely excised. No macroscopic lesions were identified in the liver, and a routine biopsy of the left lateral lobe was performed using a 6 mm punch biopsy. Hemostasis was achieved by the local application of an absorbable gelatin sponge. No other suspected metastatic or invasive lesions were found intraoperatively. Routine closure of the abdomen, subcutaneous sutures, and cutaneous sutures were performed.

**Figure 3:**
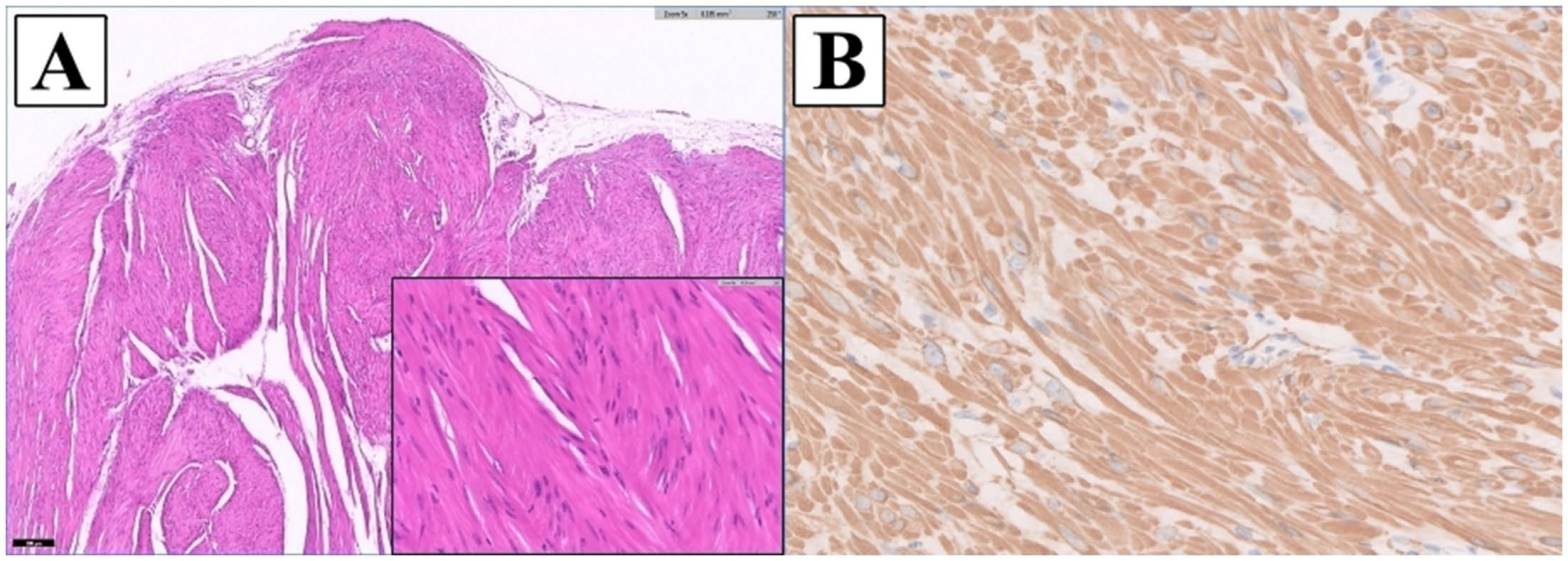
Histopathologic images of the gallbladder tumor. **(A)** The mass was composed of interlacing streams and bundles of spindle cells with indistinct borders, moderate eosinophilic fibrillar to vacuolated cytoplasm, and oval to elongate nuclei with coarsely stippled chromatin and small, variably distinct nucleoli. Mild anisocytosis and anisokaryosis were observed (H&E: x5 magnification; inset: x40) **(B)** The neoplastic cells were diffusely immunopositive for a smooth muscle actin stain confirming this mass as a leiomyosarcoma (IHC; Smooth muscle actin: x40 magnification).

Postoperative histopathologic examination confirmed a spindle cell tumor (IDEXX Laboratories, Inc., United States) (Figure 3). An unencapsulated, multilobular, moderately to densely cellular neoplasm, supported by a dense fibrous to myxomatous stroma, was arising from the tunica muscularis and replacing a large portion of the gallbladder wall. The tumor was composed of interlacing streams and bundles of cells, with indistinct cell borders and moderate eosinophilic fibrillar to vacuolated cytoplasm, and oval to elongate nuclei with coarsely stippled chromatin and small variably distinct nucleoli. Increased cellularity, mild peripheral infiltration at the serosal surface, and rare mitotic figures were observed. The tumor was separated from the surgical margin by a thin capsule and no neoplastic cells were present at the bile duct surgical margin. For definitive diagnosis, immunohistochemical analysis was performed. The neoplasm was diffusely immunopositive for smooth muscle actin stain confirming this mass as a leiomyosarcoma. The liver biopsy revealed mild vacuolar hepatopathy, mild to moderate centrilobular cytoplasmic brown pigment with scattered rare pigment granulomas, and mild bile duct hyperplasia.

The patient was discharged after 3 days without any complications. Adjuvant chemotherapy with doxorubicin was considered (2), however, the owner declined further treatment. No evidence of local recurrence or distant metastasis was found on thoracic radiographs and abdominal ultrasonography performed every 3 months. At 18-month follow-up, the dog was still alive and remained asymptomatic.

## 3 Discussion

Leiomyosarcoma is a malignant tumor originating from smooth muscle cells and classified as a type of soft tissue sarcoma (14). Common primary sites of leiomyosarcoma in dogs include spleen, gastrointestinal tract, and liver (15). Except for tumors that occurred in the liver, the prognosis for surgical treatment was relatively good with a median survival time of 10 months. For primary abdominal visceral soft tissue sarcoma in dogs, mitotic index of less than 9 was a significantly better prognostic factor, and when the grading system of cutaneous soft tissue sarcoma was applied to these tumors, dogs with grade I had longer median survival times than dogs with grade II, III (16). Surgical margin status is a critical determinant of local recurrence, as dogs with incomplete margins were reported to be 10.5 times more likely to develop local recurrence than those with complete margins (17). In human gallbladder cancer, the presence of microscopic residual tumor and lymph node metastases significantly impacts the prognosis associated with reduced disease-free interval and overall survival times (3). In this case, postoperative histopathology confirmed complete resection of the tumor (R0), and no metastasis to other organs was identified. The tumor was relatively well differentiated with mitotic count (per 2.37 mm^2^) of 1, and no necrosis was observed. The low histological grading of tumor and complete surgical resection may explain the absence of recurrence or distant metastasis observed during 18-month follow-up.

Ultrasonography is a commonly used diagnostic modality for the evaluation of gallbladder disease, however, differentiating malignant from benign lesions remains challenging due to overlapping sonographic features. In dogs, gallbladder tumors typically appeared as sessile masses with variable sizes, echogenicity, and gallbladder wall thickening. No specific ultrasonographic findings were identified to correlate with malignancy or to differentiate between different histopathologic types of tumors (4). In this case, ultrasonography revealed the sessile mass with heterogeneous echogenicity, no vascularization on Doppler imaging, and no gallbladder wall thickening. These findings are consistent with the nonspecific ultrasonographic features reported in canine gallbladder disease. Similarly, in humans, radiologic findings of an enlarged gallbladder with polypoid mass protruding into the lumen and irregularly thickened walls have been reported, but it remains difficult to distinguish leiomyosarcoma from other tumors with diagnostic imaging (1). Although advanced imaging techniques, such as ^18^F-fluoro-2-deoxy-D-glucose positron emission tomography/computed tomography or magnetic resonance imaging, have improved the diagnostic accuracy of sarcomas (1), further studies on the imaging evaluation of gallbladder tumors are needed.

ICG NIRF has shown feasibility in visualizing the biliary structures in cholecystectomy. In human study, ICG applications resulted in visualization rates of the cystic duct up to 86.5% before dissection of Calot’s triangle and increased to 96.5% post-dissection (18). Furthermore, ICG-NIRF cholangiography enables non-invasive, real-time visualization of the biliary anatomy, facilitating accurate assessment of ductal patency and minimizing the risk of iatrogenic bile duct injury during surgery (12). Impaired bile flow results in filling defects on cholangiography, which provide indirect evidence of luminal narrowing or obstruction (12, 13). In the present case, ICG NIRF imaging clearly visualized the biliary tract except at the site of the tumor, and this filling defect was presumed to result from altered bile flow due to partial luminal deformation caused by tumor. The patency of the common bile duct was confirmed intraoperatively without additional surgical intervention. These findings suggest that ICG NIRF cholangiography may be a feasible adjunct for indirect luminal evaluation to reduce surgical complications and operative time. However, since the protocol used in this case optimized for cholangiography, further studies are needed for optimal protocols based on the enhanced permeability and retention effect of ICG for tumor visualization.

Guidelines regarding dosage and administration of ICG are still under discussion. In a systematic review in humans, the dosage of ICG used for biliary tract visualization varied between a fixed dose of 2.5 mg and a body weight-based dose of 0.05 mg/kg with both dosages resulting in high visualization rates. The timing of ICG administration was consistent across studies, typically administered 45–60 min prior to surgery (18). In one study in dogs with gallbladder mucocele, 0.25 mg/kg of ICG administered at least 45 min before surgery provided successful visualization of the biliary tract and confirmed common bile duct patency in all cases (13). Based on previous studies, 0.05 mg/kg of ICG was administered intravenously to the dog 45 min before surgery. This resulted in adequate biliary tract visualization by NIRF imaging during cholecystectomy. After intravenous ICG administration, no adverse reactions, mild or severe, were observed.

In conclusion, leiomyosarcoma should be considered as a differential diagnosis for dogs with suspected gallbladder masses. In addition, ICG NIRF imaging during cholecystectomy can be considered as a surgical option as it provides real-time anatomic visualization and improved surgical outcomes. Because gallbladder tumors have been rarely reported in dogs, further studies are needed to understand the pathophysiology, diagnosis, optimal treatment and prognosis.

## Data Availability

The original contributions presented in the study are included in the article/supplementary material, further inquiries can be directed to the corresponding author.
